# Successive Dropsies of the Amnion Always Specific

**Published:** 1884-01

**Authors:** James Dorland

**Affiliations:** Milwaukee, Wis.


### Selections.

SUCCESSIVE DROPSIES OF THE AMNION ALWAYS SPECIFIC. By JAMES DORLAND, M.D., Milwaukee, Wis. Read before the Canada Medical Association, at Kingston, September, 1883.

CASE I.—Mrs. D., aged 30; full habit of body, and apparently healthy. Was called to attend her for the first time, in her fifth confinement, July 16, 1880. Found the os fully dilated, and membranes very tense, and bulging with fluid. Externally, found the abdomen high up, interfering somewhat with respiration, the skin very tight, and palpation giving evidence of a large amount of fluid. Ruptured the membranes, and it was followed by a great gush of water, then the child, and another large quantity of fluid. The amount was between eight and ten quarts, but certainly not less than the former. The fœtus proved to be about seven months old, and had been dead about 24 hours. She said it was not an unusual amount of water, as in all her previous confinements she had about the same quantity. Had been married to a sailor eight years. Could give no reason why so much water would form. The other four children were still-born at four, six and seven months. Had been treated for it at various lake ports, but derived no benefit. Knowing the habits of sailors, I began to look about for a cause, but both of them denied ever having any specific disease. I was not fully convinced, and determined, when she again became pregnant, to put her on specific treatment.

About the 1st of January, 1881, finding her in that condition, I began treatment, and continued it thoroughly until she had passed the seventh month and was well on toward the eighth. At the ninth month, September 3, I delivered her of a 13 lb. girl, which was perfectly healthy and is alive to-day, and has shown no traces whatever of syphilis. Did not allow the mother to nurse it. There was no dropsy. She again became pregnant, and, satisfied that if she were to blame, the previous vigorous treatment would be sufficient, I only gave her a couple of bottles of medicine.

August 3, 1882, I delivered her of an 11 lb. boy, healthy, and well developed. It has since died, but from no disease connected with syphilis. There was a slight increase of fluid over the former, not amounting to dropsy.

She became pregnant in the following November, but I did not see her until I was called, August 5, 1883, to attend her. She complained of not feeling as well as with the two former, and had felt no life for two days. Upon examination, found dropsy of the amnion, and thought the child would be dead, as I was not certain I could detect the fœtal bruit. About five quarts of water came away, and she was delivered of an 8 lb. girl, which presented marks of syphilis on its hands, feet, and several portions of the body. She now admitted having had some sores, bnt stated they were after marriage. The child was alive, and was at once put on specific treatment.

CASE II.—Mrs. P., aged 25, of short stature, but well developed; married to her second husband; the mother of one boy by her first, aged 15, strong and healthy. Had six children by her present husband, all born dead, or died shortly after, within three or four weeks. I saw the last one November 1, 1880; it was about three weeks old, and had well marked syphilis, and died about three days after. Both parents strenuously denied ever having had any specific disease, but the previous history of the mother, and the loose character of the husband, led me to suspect him as the culprit. With all her children by the last husband, there had been an abundance of water, anu the midwife said some of them had been drowned. When she again became pregnant, I put her on specific treatment, and delivered her, October 4, 1881, of a 9 lb. girl, and at full time, alive, with no traces of syphilis, and about the ordinary quantity of liquor amnii. Did not allow her to suckle it. The child is still alive, and healthy.

A short time after, she became pregnant, and, not believing what was told her, refused to take specific treatment. When labor came on, was sent for; found the os fully dilated, a large bag of water protruding into the vagina, and the abdomen very tense. Ruptured the membranes, and succeeded in collecting 10 quarts of water. The child was dead. The delivery was August 10, 1882. In November she again became pregnant, and took medicine as directed, and on August 11, 1883, I delivered her of a fine healthy child, without any dropsy. She had felt much better than when carrying her previous child.

CASE III.—Mrs. H., aged 25; spare habit of body, but wiry; the mother of four children. The first two only lived a few weeks after birth. I did not attend her with either of them. Had treated the father for syphilis. She stated that when they were born a great quantity of water came away; supposed that was natural, until after her next was born. The midwife stated there were about six or seven quarts of fluid. With her third and fourth I attended her, and kept her on specific treatment during gestation. They were both fine healthy children, but one of them has since died from convulsions. At neither of her last confinements was there any undue collection of fluid. The father had also, in the meantime, taken a further specific course.

CASE IV.—Mrs. H., aged 17; married at 14; of short stature, but strong and healthy; the mother of three children, two of them being now dead. Had treated her husband for syphilis before marriage, but he did not take a very thorough course. When pregnant with her first child, advised specific treatment, but they would not listen to it.

August 22, 1881, delivered her of a seven months' fœtus, which was dead. The cord was only 8 inches long. On rupturing the membranes, about seven quarts of water came away. The child had only been dead a few hours, probably since she had a chill. She soon became pregnant again, but still persisted in not taking any medicine.

July 16, 1882, was called; found the membranes ruptured, and about half the liquor amnii in a pail. It measured six quarts. When the water flowed freely, she would get up and go to the pail, and wait until the pain was over. I do not think it any exaggeration to state that as much was lost on the bed-clothes and floor. When the child was born, about another quart came away, making 12 or 13 quarts altogether. Contrary to my expectations, the child was alive, and lived two weeks before death, presenting the characteristic old man appearance of syphilis.

When she again became pregnant, consented to take medicine, and it was pushed thoroughly. About ten days before labor she began to enlarge quite fast, and upon examination, found more fluid than normal. Had stopped all treatment for about three weeks previous; now, however, I again began giving potassium iodide ʒi per day, which acted on the bowels quite freely. I continued it until the birth of the child. About the fifth day after taking the pot. iod. the movements of the child became stronger, and the tenseness gave way.

July 6, 1883, I delivered her of a 10 lb. boy, with only slight traces of syphilis, and no undue amount of fluid.

CASE V.—Mrs. S., aged 41; short and robust; the mother of nine children. The first was born in Scotland, the second in England, five in Greece, the eighth in England, and the ninth in the United States. Generally noticed that she began to enlarge quite rapidly a fortnight or six weeks before the birth of her children. First child still-born, with an immense quantity of fluid; fifth, a large amount, but the child was alive, and died two weeks after; eighth child only lived 20 minutes, and she thought the death was due to too much fluid. I was called to attend her with the ninth; found the os fully dilated. I ruptured the membranes and collected six quarts of water; not more than a pint escaped. The child, a male, weighed 13 lbs., and died 15 minutes after birth. The cord was 12 inches long, and fully an inch in diameter. She was confined March 23, 1883. Could get no specific history from either parent.

CASE VI.—Mrs. W., aged 38; spare habit of body; the mother of five healthy children by her first husband. Her second one had three by his first wife, all healthy. He contracted syphilis, and gave it to this one. They paid very little attention to medicines prescribed; only when some external manifestations were present, and painful, they would use some local application until relieved. During this time, which was in 1881, she became pregnant, and, contrary to advice, stopped treatment. At the eighth month she had some severe pains, and sent for me. Upon examination, found the os dilated so as to admit the tip of the finger. The membranes were as tense as they should be during a severe pain. A great change had taken place in her abdomen, it being enormously distended. It was symmetrical, and pushed up the diaphragm so that respiration was very much interfered with. Expression anxious. Said the skin felt as though it would burst. Gave her an anodyne. The following day she had a chill, and four days after, labor came on. I arrived a few minutes after the membranes had ruptured, and found the bedding and carpet saturated with water. The patient was in an anxious frame of mind; felt sure death was approaching, as, to use her own words, she had collapsed. Said she had passed fully 24 quarts of water, a great gush following and during a second pain. I soon delivered her of a still-born child. At the lowest calculation, there could not have been less than 20 quarts, and I firmly believe, could it have been collected, it would have reached 30, for I have never seen such a flood in a parturient chamber.

### REMARKS.

CASE I.—This patient, after having five still-births, all with dropsy, was able to have, after one thorough course, three children in succession, all born alive—the first two without dropsy, and without any sign of syphilis; the last with dropsy and a showing of syphilis, but still alive, and with fair prospects of living. But the last case also shows that some of the effect produced by specific treatment is beginning to wear off. After this last one, I received my first intimation from her that she had ever been diseased, but I had long since found out I was right from a sister of hers who came to the city later and entered a house of prostitution.

CASE II.—This is also a very valuable one, for here we have six cases of dropsy following in succession, the seventh, under specific treatment, being born without, and perfect in every respect, and free so far from any sign of syphilis. The next, or eighth child, with no treatment whatever, again born with dropsy, and dead. The ninth, after quite a thorough course, was also born alive, and without dropsy or any sign of syphilis. The husband proved to be the guilty party in this case, and probably that was the reason the first thorough treatment was not sufficient to tide the next one over till full time.

CASE III.—Although I did not see the two children that were born with dropsy of the amnion, the report is perfectly reliable, and I think to the specific course of treatment can be attributed the next two natural births. The father had also, after the birth of the second child, taken further treatment.

CASE IV.—Here we had two cases of dropsy, the husband known to be syphilitic; the third, after the mother had been treated, born alive and with no dropsy, but still with slight traces of syphilis on the palms of the hands and soles of the feet. The father in this case had a very severe attack, and took but little medicine, so that his blood is in a much worse state than any of the others. It is to that I assign the reason of the thorough treatment of the mother not removing every trace of syphilis from the child.

CASE V.—Although not mentioned in my report, all the nine children that were born had dropsy of the amnion, the ones I specified being the most excessive. Although no history from father or mother that would point to syphilis, the condition of the living children, their stunted growth, saw-edged teeth, general appearance, and the illness of one of them, for which no cause was apparent, and which had not yielded to other forms of treatment, and which potassium iodide readily cured, leave scarcely a doubt in my mind but that the father or mother has had syphilis, or is the victim of an inherited taint.

CASE VI.—Although no direct bearing on successive cases, the fact that there could be no doubt as to the cause of the dropsy and the excessive amount of fluid, led me to report the case.

These are the facts I have to lay before you in support of my theory that successive dropsies of the amnion are always specific; facts that cannot be obtained from our lying-in hospitals, but from private practice, where attendance on a family has extended over a number of years, and where you have the time and patience to ferret out the cause of diseases from many who do not care to have you know their previous misfortunes, or do not inform you for fear of further complications. In hospitals, we seldom see the same patient twice, hence the conclusions arrived at there would be of no use either for or against this theory. That syphilis has caused it, has more than once been advanced, but generally denied, and I think the Germans are the only ones who put much faith in it. That syphilitic children are born without dropsy often happens, for no doubt most of you have seen such cases. These cases of dropsy could not have arisen from any other cause, for there is no history in any of the cases of any inflammation of the amnion, for not one of them suffered from the least bit of pain, but only discomfort, after the fluid had accumulated. Very few of them ever had a chill, and in those the dropsy was present before that time, and the chill only seemed to mark the death of the infant, and not always that. None of them ever had any general anasarca during gestation, and their kidneys always seemed to be fairly active. It could not have been disease of the heart and kidneys of the infant, because some of them were born at such an age, as the one at four months, with about four quarts of fluid, that it would be an utter impossibility; besides, many of them were born alive, and are still alive, and, in most of the cases, even at full term, the dropsy came up in such a short time that the kidneys of no infant could have secreted the amount. I was sorry that I could obtain no post-mortems of the cases I attended, but even had I found hypertrophy of the heart or kidneys, I should have placed no stress upon it, for I do not believe it could cause it, nor that we have any facts that point in that direction. The death of the fœtus and osmosis could not have produced it, for the same reason stated above, that many of them were alive, and lived some time after birth. It has seemed to me that it would be impossible to have a dropsy except from systemic infection producing changes in the amnion, or following a debilitating disease, as latent pleurisy often does; but that all these cases were in good health shows that it was not due to the latter, but to a poison which can lurk for years in the system, and, as far as outward signs go, give no evidence of its presence. Such a poison is syphilis, and I believe that if not the only cause, it is the one *par excellence*, and the only cause of successive cases.

Cases 1 and 2 would, I think, have been sufficient for my purpose, but the others were added because in unity there is strength. These cases all happened in mothers who spent many hours in grief because of the loss of their children and their inability to have them born alive; and if to any such among your patients relief is brought, I shall feel amply repaid.—*Canada Medical and Surgical Journal.*

---

### A SUCCESSFUL INJECTION OF A SOLUTION OF SALT IN ACUTE ANÆMIA.

The death in hæmorrhage is not directly caused by the loss of blood, but by the immobility of the remaining blood corpuscles. If a fluid is injected into the vessels and thus the tonus of the vessels restored, the individual will recover. It has been proved by Ott that in very severe hæmorrhages the number of red blood corpuscles and the quantity of albumen was normal a few weeks after an injection of salt solution. The following case will prove the truth of this theory. A woman who had been delivered from placenta prævia with severe hæmorrhage was found moribund and without radial pulse. An injection of one and a half liters of a 6 per cent. solution of salt with three drops of a solution of caustic soda was made into the vena mediana. After three minutes the radial pulse was perceptible, and the patient made a deep respiration. After five days the woman was comfortable. The injection has to be made into a vein, as the veins can easily be detected in an anæmic person, and as there has been gangrene after injection into an artery.

### TREATMENT OF POLYURIA.

℞—Pure glycerine........30 grams.
Citric or tartaric acid..2 grams.
Water...............500 grams.

M. Sig. a tablespoonful (or two) every hour. Under the influence of this treatment the urea emitted in 24 hours diminishes 6 to 7 grams.—*Revue Thérap. Medico-chirurgicale.*

